# Ichthyological collection of the Museu Oceanográfico D. Carlos I

**DOI:** 10.3897/zookeys.752.20086

**Published:** 2018-04-23

**Authors:** Ana Serra Silva, Maria Pitta Groz, Paula Leandro, Carlos A. Assis, Rui Figueira

**Affiliations:** 1 Aquário Vasco da Gama, Rua Direita do Dafundo, 1495-718, Cruz Quebrada-Dafundo, Portugal; 2 Division of Biosciences, University College London, Gower Street, London WC1E 6BT, UK; 3 Department of Life Sciences, Natural History Museum, Cromwell Road, London SW7 5BD, UK; 4 Institute of Zoology, Zoological Society of London, Regent’s Park, London NW1 4RY, UK; 5 Universidade de Lisboa, Faculdade de Ciências, Departamento de Biologia Animal, Campo Grande, 1749-016 Lisboa, Portugal; 6 MARE–Marine and Environmental Sciences Center, Campo Grande, 1749-016, Lisboa, Portugal; 7 CIBIO/InBIO-Centro de Investigação em Biodiversidade e Recursos Genéticos, Universidade do Porto, Vairão, Portugal; 8 CEABN/InBio, Centro de Ecologia Aplicada “Professor Baeta Neves”, Instituto Superior de Agronomia, Universidade de Lisboa, Tapada da Ajuda, 1349-017 Lisboa, Portugal

**Keywords:** Natural History collection, D. Carlos I, Animalia, Myxini, Petromyzonti, Elasmobranchii, Holocephali, Actinopterygii, Occurrence, Portugal

## Abstract

The collection of the Museu Oceanográfico D. Carlos I is a historical specimen, instrument, and document collection that has been housed at the Aquário Vasco da Gama since 1935. The collection is largely the result of several scientific campaigns conducted by Dom Carlos de Bragança between 1896 and 1907. Specifically, the ichthyological collection consists of 675 surviving catalogue records of specimens caught, acquired or offered to D. Carlos I between 1892 to 1907, and includes the type specimen for *Odontaspis nasutus* Bragança, 1904 (junior synonym of *Mitsukurina owstoni* Jordan, 1898), along with several specimens of deep sea species. All specimens were captured in coastal Portuguese waters, and were preserved in alcohol, formalin, or mounted.

## Introduction

Dom Carlos I, king of Portugal and the father of Portuguese oceanography (Saldanha 1997, 2002), was an avid naturalist. He was heavily influenced by his love of the sea, a love imparted by his father, D. Luis I, and also by the scientific explorations of his friend Prince Albert I of Monaco (Carpine-Lancre and Saldanha 1992, Ceríaco 2014). These influences, along with the many foreign scientific campaigns that crossed Portuguese waters and the impact that greater oceanographic knowledge could have on Portuguese fisheries, inspired D. Carlos I to organise a series of oceanographic campaigns to study the bathymetry and fauna of the Portuguese coast (Bragança, 1897b). These campaigns, undertaken between 1896 and 1907, resulted in many manuscripts, but only four formal publications (Bragança 1897b, 1899b, 1902b, 1904b), and a magnificent collection of marine animal specimens, some of which were displayed in national and international exhibitions (Saldanha 1997).

The writings of D. Carlos I include eleven detailed annual reports of his oceanographic campaigns (Bragança 1896, 1897a, 1898, 1899a, 1900, 1901, 1902a, 1903, 1904a, 1905, 1907), formally published in 1897 and 1902 (Bragança 1897b, 1902b), one publication on the tuna fisheries in the Algarve (Bragança 1899b), and one on the shark species captured along the Portuguese coast during the 1896-1903 campaigns (Bragança 1904b).

In his reports, D. Carlos I described, in great detail, the programme and objectives of each campaign, the sampling stations and materials used in each, their depth and benthic characteristics, and listed the specimens collected (Bragança 1896, 1897a, 1898, 1899a, 1900, 1901, 1902a, 1903, 1904a, 1905, 1907). In his “Esqualos obtidos nas costas de Portugal durante as campanhas de 1896 a 1903” (Bragança 1904b), D. Carlos I catalogued all the shark species captured, their geographical and bathymetric distribution, their systematic position, with the accepted scientific name and synonyms, and their vernacular names (in both Portuguese and French). D. Carlos I also described the morphological characteristics, length and coloration, stomach contents, and the economic uses for each species. In addition, D. Carlos also provided identification keys and an ecological classification for the collected species. As well as this published work, D. Carlos I also left many manuscript notes with similar information on other fish groups, possibly intended to serve as basis for other publications (Saldanha 1997).

The collection of the Museu Oceanográfico D. Carlos I has been housed at the Aquário Vasco da Gama (AVG) since 1935, but it comprises only a small fraction of the material collected by D. Carlos, as many specimens were lost between the king’s assassination, in 1908, and the collection’s transfer to the AVG (Nobre 1935, Carpine-Lancre and Saldanha 1992). The collection is a result of the king’s fastidious work, and exploration of Portugal’s distinctive underwater geography. Underwater canyons over 1000 m deep are as close as 5 nautical miles (9.26 Km) from the coast (Instituto Hidrográfico 2008), resulting in the occurrence of bathypelagic faunal assemblages close to shore. These geographical conditions facilitated the capture of a large number of deep-sea species, including the holotype of *Odontaspis nasutus* Bragança, 1904 (junior synonym of *Mitsukurina owstoni* Jordan, 1898).

The wider collection of the Museu Oceanográfico D. Carlos I is made up of ichthyological, mammalian, ornithological, reptilian and a wide variety of invertebrate specimens, along with scientific instruments, and a rich scientific library that includes the king’s manuscripts and copies of his published works. This collection is of incalculable historical and scientific value as it is one of the few surviving royal Natural History collections in Portugal, most of which were destroyed in a fire at the Museu Bocage in 1978 (Alves et al. 2014, Ceríaco 2014). Of this material, ichthyological specimens are the most numerous (675 records), and have the most data associated with them, while also being extremely diverse and rich in rare deep-sea specimens.

Given the historical value of the collection and its wide faunal assemblage, a dataset of the ichthyological specimens housed at the Museu Oceanográfico D. Carlos I was made available on the Global Biodiversity Information Facility (GBIF) data portal. This dataset established the first records of at least 184 species in Portuguese waters as far back as the period between 1892 and 1907.

The objectives of the present paper are: (1) to present the existence and the composition of the ichthyological collection of the Museu Oceanográfico D. Carlos I, which comprises 675 records, captured between 1892 and 1907; and (2) to emphasise its importance, not only because of its historical value, but also due to its diversity and the rarity of some of the specimens within it. We also provide the historical context, and a summary catalogue of the taxa in the collection, and highlight some notable specimens.

## General description

The dataset is comprised of the ichthyological specimens from the collection of the Museu Oceanográfico D. Carlos I. These specimens consist of 675 catalogued records, composed of 5 classes, 35 orders, 119 families, 196 genera, and 236 species. There are between 590 and 600 specimens preserved in alcohol and formalin, and between 75 and 90 mounted specimens, collected between 1892 and 1907. Many specimens have accompanying collection data in the king’s manuscripts. The records include the holotype of *Odontaspis nasutus* Bragança, 1904 (junior synonym of *Mitsukurina owstoni* Jordan, 1898), an exquisitely preserved *Nemichthys scolopaceus* Richardson, 1848, and a mounted specimen of *Centrophorus lusitanicus* Barbosa du Bocage & de Brito Capello, 1864.

### Project details

**Project title**: Revisão Taxonómica e Consolidação dos Catálogos das Coleções Ictiológicas do Aquário Vasco da Gama

**Funding**: No funding was required or used for this study.

### Taxonomic coverage

**Figure 1.**
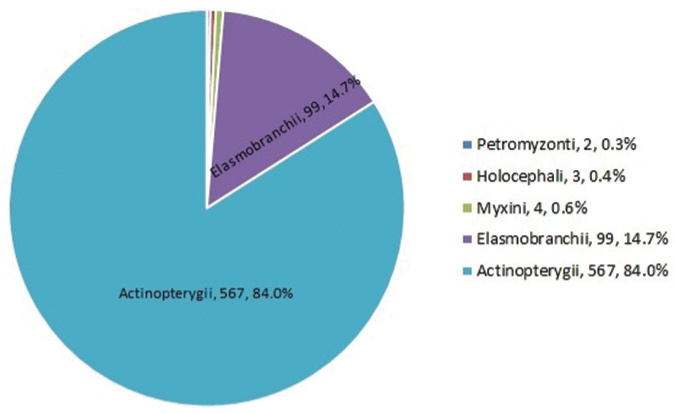
Number and percentage of the classes represented in the dataset.

**Figure 2.**
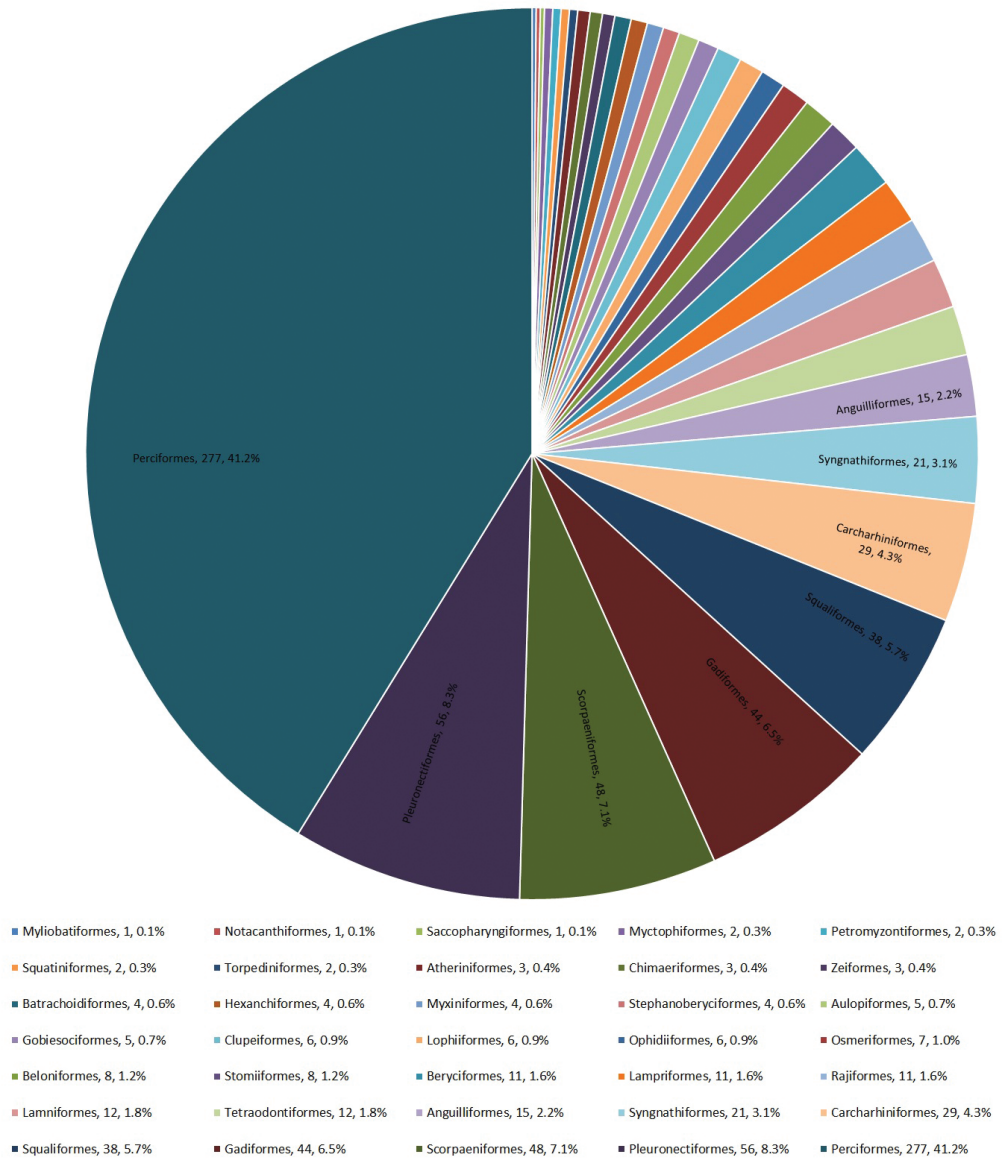
Number and percentage of the orders represented in the dataset. Only the orders with at least 15 specimens are labelled.

**Figure 3.**
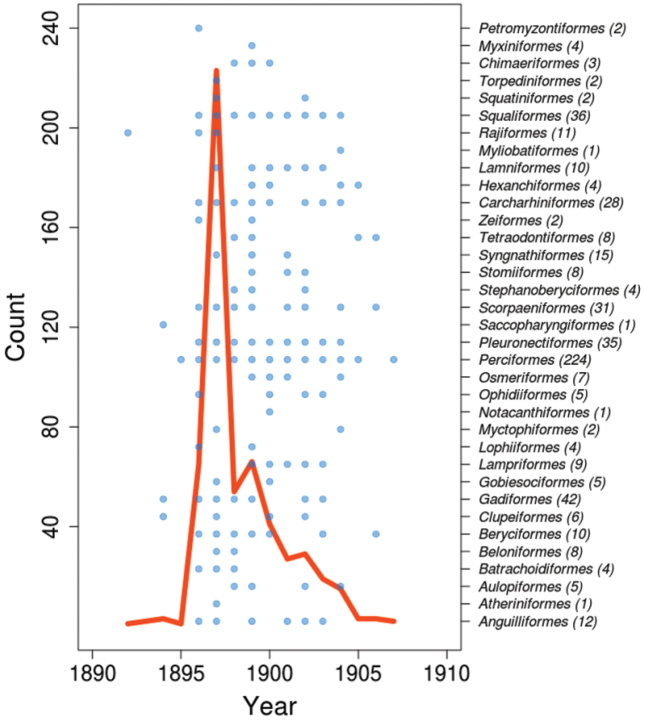
Temporal profile of the sampling years of the specimens held in the ichthyological collection. Blue dots represent sampling years for each order, for which, in parentheses, the number of specimens is provided. The red curve shows the number of specimens collected per year.

**General taxonomic coverage description**: The collection comprises representatives of the classes Myxini, Petromyzonti, Elasmobranchii, Holocephali and Actinopterygii, with actinopterygians representing over 80% of all specimens (Figure 1). The classes Myxini, Petromyzonti, and Holocephali are each represented by a single species, with a varying number of specimens for each species. There are 30 genera and 38 species of elasmobranchs, from eight orders and 23 families. There are 163 genera and 192 species of actinopterygians, from 24 orders and 94 families, including Perciformes, which corresponds to 41% of the entire dataset (Figure 2). Figure 3 shows the temporal sampling profile of the specimens, and the number of specimens per order. Of 675 records, 652 are identified to the species or subspecies level, representing a total of 196 genera and 236 species.

### Taxonomic ranks

**Kingdom**: Animalia

**Phylum**: Chordata

**Class**: Actinopterygii, Elasmobranchii, Holocephali, Myxini, Petromyzonti

**Order**: Anguilliformes, Atheriniformes, Aulopiformes, Batrachoidiformes, Beloniformes, Beryciformes, Carcharhiniformes, Chimaeriformes, Clupeiformes, Gadiformes, Gobiesociformes, Hexanchiformes, Lamniformes, Lampriformes, Lophiiformes, Myctophiformes, Myliobatiformes, Myxiniformes, Notacanthiformes, Ophidiiformes, Osmeriformes, Perciformes, Petromyzontiformes, Pleuronectiformes, Rajiformes, Saccopharyngiformes, Scorpaeniformes, Squaliformes, Squatiniformes, Stephanoberyciformes, Stomiiformes, Syngnathiformes, Tetraodontiformes, Torpediniformes, Zeiformes

**Family**: Alepisauridae, Alepocephalidae, Alopiidae, Ammodytidae, Anguillidae, Antennariidae, Argentinidae, Atherinidae, Aulopidae, Balistidae, Batrachoididae, Belonidae, Berycidae, Blenniidae, Bothidae, Bramidae, Callionymidae, Caproidae, Carangidae, Carapidae, Carcharhinidae, Caristiidae, Centracanthidae, Centriscidae, Centrolophidae, Centrophoridae, Cepolidae, Cetorhinidae, Chaunacidae, Chiasmodontidae, Chimaeridae, Chlamydoselachidae, Citharidae, Clinidae, Clupeidae, Congridae, Cottidae, Cynoglossidae, Dalatiidae, Diodontidae, Echeneidae, Echinorhinidae, Epigonidae, Etmopteridae, Gadidae, Gempylidae, Gobiesocidae, Gobiidae, Gonostomatidae, Haemulidae, Hexanchidae, Himantolophidae, Labridae, Lamnidae, Lampridae, Lophiidae, Lotidae, Macrouridae, Melamphaidae, Merlucciidae, Mitsukurinidae, Molidae, Moridae, Moronidae, Mugilidae, Mullidae, Muraenidae, Myctophidae, Myliobatidae, Myxinidae, Nemichthyidae, Nomeidae, Notacanthidae, Ophichthidae, Ophidiidae, Oxynotidae, Peristediidae, Petromyzontidae, Phycidae, Polyprionidae, Pomacanthidae, Pomacentridae, Pomatomidae, Pseudotriakidae, Rajidae, Regalecidae, Rhinobatidae, Saccopharyngidae, Sciaenidae, Scomberesocidae, Scombridae, Scophthalmidae, Scorpaenidae, Scyliorhinidae, Sebastidae, Serranidae, Soleidae, Somniosidae, Sparidae, Sphyraenidae, Sphyrnidae, Squalidae, Squatinidae, Sternoptychidae, Stomiidae, Stromateidae, Synaphobranchidae, Syngnathidae, Tetraodontidae, Torpedinidae, Trachichthyidae, Trachinidae, Trachipteridae, Triakidae, Trichiuridae, Triglidae, Uranoscopidae, Xiphiidae, Zeidae

### Spatial coverage

**Figure 4.**
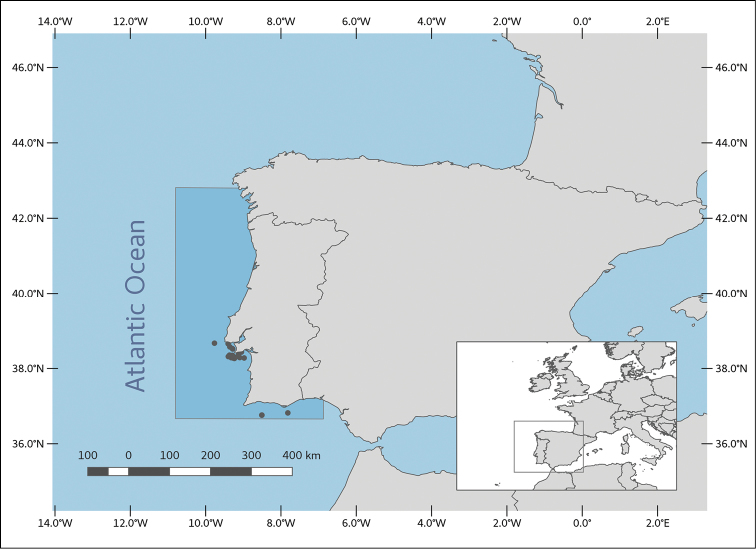
Geographic area covered by the collection (in darker blue). Plots indicate sites for the 29 georeferenced records.

**General spatial coverage**: The bulk of the specimens were collected at the mouth of the rivers Tagus and Sado, and in the bays of Cascais and Sesimbra, usually within 50 nautical miles of the coast. The collection also features several specimens caught on the Algarve coast, a handful of specimens from the coast north of the Cabo da Roca, and one specimen tentatively identified as being from the Azores. The sampling depths range from surface level to 1875 m deep. Most specimens were captured within the polygon defined by the following coordinates: 36°42'14"N and 42°17'38"N latitude; 10°43'22"W and 6°11'45"W longitude (Figure 4).

### Temporal coverage

July 18, 1892–June 8, 1907

### Natural collections description

**Collection name**: Coleção do Museu Oceanográfico D. Carlos I

**Collection identifier**: bf52e001-eb74-421e-9d51-157d6eb5a358

**Specimen preservation method**: Alcohol, Formalin, Mounted

**Curatorial unit**: Between 590 and 600 jars, between 60 and 70 mounted specimens, and 16 jaws

## Methods

**Method step description**: The ichthyological collection of the Museu Oceanográfico D. Carlos I was first catalogued for publication by Gonçalves (1942). That publication consisted of the first taxonomic check list since the collection moved to the AVG (Gonçalves 1942). Between 1942 and the present day only minor synonymic revisions have been made and noted in the card catalogue, with the exception of the Blenniidae, which were re-identified and described by Almeida (1981). More recently, an electronic catalogue was created as an Excel (Microsoft, Washington, USA, 2010) spread sheet, without systematic synonymy revision as it encompassed all the collections and taxa housed in the AVG.

The first step of the current revision project was to isolate ichthyological records from the electronic catalogue, and check them for synonymy using both FishBase (Froese and Pauly 2017) and the Catalogue of Fishes (Eschmeyer et al. 2017). The revised electronic catalogue was then cross-checked with the card catalogue and Gonçalves (1942) for collection date and location, depth of collection, and species name. If species names did not match the currently accepted name or the synonym used in the first electronic catalogue, they were re-checked for synonymy. When the card and electronic catalogue both had accepted, but different, species names, or the synonyms did not belong to the same valid taxon, the records were flagged for further identification.

Once catalogue crosschecking was complete, specimen labels were compared to the final electronic catalogue, both to confirm the information and to check the location of the specimens. The crosschecking process also identified two mislabelled elasmobranch specimens, which were identified using Compagno’s Sharks of the World (1984). When mismatches were found, the information in the catalogue was substituted with the information from the specimen labels, including species names.

The last step in the creation of the electronic catalogue was to resolve naming inconsistencies. First, the species identification present on labels was checked for synonymy. Once valid names had been identified, the species range for each valid taxon was ascertained using FishBase (Froese and Pauly 2017). If one of the ranges was incongruent with the collection locality, the concordant species name was chosen for each record. If both ranges were congruent with the collection locality, the specimens were re-identified by the authors.

For publication purposes, the records pertaining to the collection of the Museu Oceanográfico D. Carlos I were extracted from the electronic catalogue and transformed into a DarwinCore compatible Excel (Microsoft, Washington, 2016) spread sheet. The dataset was enriched with collection data taken from the king’s records of his oceanographic campaigns (Bragança 1896, 1897a, 1898, 1899a, 1900, 1901, 1902a, 1903, 1904a, 1905, 1907). The additional data included navigational bearings, depth ranges, and sampling methods.

**Study extent description**: The specimens belonging to D. Carlos’s ichthyological collection can be divided in three non-taxonomical groups: specimens caught during the king’s oceanographic campaigns, specimens caught by the king outside the campaigns, and those offered to the king. These groups vary in collection data completeness, with the specimens captured during oceanographic campaigns generally having the most information, including navigational bearings and collection depths, and those offered to D. Carlos having the least information attached to them, some have only the collection locality or purchase location. However, all specimens were captured in Portuguese coastal waters, and those caught by the king were captured mostly in the area between the Cabo da Roca and Setúbal, and some in the Algarve. The specimens were captured year-round, between 1892 and 1907, but the oceanographic campaigns were usually held in the spring and summer months, from 1896 to 1907.

**Sampling description**: The specimens were caught using a variety of fishing nets, lines and traps (côvo). The line fishing methods included longline fishing (espinhel) and angling. The fishing nets used range from bottom and midwater trawls to dragnets. Traditional fishing techniques may also have been used, as some specimens were bought in markets or offered by fishermen, but there is no specific additional information in these cases. Nonetheless, there is record of at least one specimen being harpooned.

**Quality control description**: The validity of species’ names was checked using both FishBase (Froese and Pauly 2017) and the Catalogue of Fishes (Eschmeyer et al. 2017). Synonymy was checked across all three catalogues, if incongruences were found between catalogues the earliest name on record was used for disambiguation. If the names used in the earliest catalogue did not resolve the nomenclatural inconsistency, geographical ranges of the species were checked and used to assign the currently accepted species name. When these steps were insufficient to identify the correct species, the authors proceeded to a more thorough re-identification of the specimens. Lastly, for mislabelled elasmobranch specimens, the identification keys in Sharks of the World (Compagno 1986) were used.

## Datasets

### Dataset description

**Object name**: Darwin Core Archive Ichthyological Collection of the Museu Oceanográfico do Rei D. Carlos I

**Character encoding**: UTF-8

**Format name**: Darwin Core Archive format

**Format version**: 1.0

**Distribution**: http://ipt.gbif.pt/ipt/archive.do?r=codc

**Publication date of data**: 2017-05-22

**Language**: Portuguese

**Licences of use**: Creative Commons Attribution (CC-BY) 4.0 License

**Metadata language**: English

**Date of metadata creation**: 2017-04-11

**Hierarchy level**: Dataset
